# Case of Persistent Facial Neuralgia, of Reflex Dental Origin, Due to an Unerupted Cuspid Tooth

**Published:** 1890-03

**Authors:** 


Case of Persistent Facial Neuralgia of Reflex Dental Origin, due to an Unerupted Cuspid Tooth.—Edward C. Kirk, D. D. S., reports a case in the *University Medical Magazine*, of a lady who had been troubled with a severe and persistent facial neuralgia, from which she had suffered for between three and four years, with but slight periods of intermission. On examination, the upper jaw was found to be edentulous,—the lower teeth back of the cuspids were also missing. Those that remained were in a perfectly healthy condition, and a careful examination of the mouth and jaws elicited nothing abnormal. The idea of an unerupted tooth occurred to the writer, and, on questioning the patient, he received the reply that she was quite positive that she had never had an eye tooth on the left side. On examination with a probe a cavity was found, and on enlarging it, an unerupted cuspid tooth was found in a horizontal position, with the apex of the root in the forward end of the antrum, and the point of the crown imbedded in the symphysis of the superior maxillary bone. The tooth was extracted, after which the persistent neuralgia has abated, and in due time a perfect cure, at least from the dental source, is to be expected.

